# Correlation of pericardial and mediastinal fat with coronary artery disease, metabolic syndrome, and cardiac risk factors

**DOI:** 10.1186/1532-429X-11-S1-O16

**Published:** 2009-01-28

**Authors:** Onn Chenn, Ijaz Ahmad, Betty Hua, Joshua A Sockolow, Igor Klem, Terrence Sacchi, John F Heitner

**Affiliations:** 1 New York Methodist Hospital, Brooklyn, NY USA; 2 Duke University Medical Center, Durham, NC USA

**Keywords:** Obesity, Diabetes Mellitus, Coronary Artery Disease, Family History, Metabolic Syndrome

## Background

Obesity and abdominal fat have been shown to correlate with coronary artery disease (CAD) and may play a role in development of metabolic syndrome (MS). The significance of pericardial adipose tissue (PAT) and mediastinal adipose tissue (MAT) is less clearly defined.

## Objective

To study the association between PAT and MAT measured by cardiac magnetic resonance with: 1) severity of CAD, 2) MS and 3) cardiac risk factors (CRF) for CAD.

## Methods

We enrolled 100 consecutive patients, 63 male, who underwent CMR for cardiac evaluation and had coronary angiogram performed within 12 months. The baseline characteristics of these patients were as follows: Eighty had hypertension (HTN), 42 had diabetes mellitus (DM), 37 had hyperlipidemia and 4 were smoker. We measured PAT and MAT on 4-chamber cine view. The surface area of fat was measured by computer analysis from free-hand region of interest (ROI) curves. The presence and the extent of CAD were measured using Duke Jeopardy Score. MS was considered positive if the patient had 3 or more of the 5 criteria. The CRF included HTN, DM, hyperlipidemia, smoking, peripheral vascular disease (PVD) and a family history of premature CAD (FH).

## Results

PAT had significant correlation with MS and HTN, but not with CAD. MAT did not have a significant correlation with either CAD or MS, but did correlate with DM, hyperlipidemia, smoking and FH. The combination of PAT and MAT correlated with MS and all the risk factors, except PVD, but did not correlate with CAD. (Data in Table 1.)

**Table 1:** Table 1

	Pericardial Fat (cm^2^)	Mediastinal Fat (cm^2^)	Total Fat (cm^2^)
CAD	8.3 {p-value = 0.317}	19.5 {p-value = 0.16}	27.7 {p-value = 0.03}
Metabolic Syndrome	9.3 {p-value = 0.0005}	18.9 {p-value = 0.19}	26.8 {p-value = 0.02}
Cardica Risk Factors			
HTN	9.5 {p. value = 0.008}	19.6 {p-value = 0.08}	29.1 {p-value = 0.03}
DM	9.9 {p-value = 0.08}	21.2 {p-value = 0.05}	31.2 {p-value = 0.03}
Hyperlipidemia	9.0 {p-value = 0.56}	20.4 {p-value = 0.01}	29.4 {p-value = 0.02
Smoking	9.2 {p-value = 0.86}	13.1 {p-value = 0.05}	22.2 {p-value = 0.27}
PVD	11.9 {p-value = 0.008}	19.5 {p-value = 0.79}	32.3 {p-value = 0.05}
FH	9.0 {p-value = 0.74}	23.2 {p-value = 0.02}	31.2 {p-value = 0.58}

## Conclusion

The combination of MAT and PAT correlates with MS and a number of CRFs but does not correlate with CAD.

